# Evaluating Food and Drug Administration approved gastrointestinal cancer drugs: clinical benefit and trial endpoints over the past two decades

**DOI:** 10.1093/oncolo/oyaf381

**Published:** 2025-12-08

**Authors:** Fares Jamal, Oudai Sahvan, Pedro Luiz Serrano Uson Junior, Jeremy C Jones, Fang-Shu Ou, Tanios Bekaii-Saab, Mohamad Bassam Sonbol

**Affiliations:** Division of Hematology/Oncology, Mayo Clinic Cancer Center, Mayo Clinic, Phoenix, AZ, United States; Division of Hematology/Oncology, Mayo Clinic Cancer Center, Mayo Clinic, Phoenix, AZ, United States; Division of Hematology/Oncology, Mayo Clinic Cancer Center, Mayo Clinic, Phoenix, AZ, United States; Division of Hematology/Oncology, Mayo Clinic Cancer Center, Mayo Clinic, Jacksonville, FL 32224, United States; Division of Clinical Trials and Biostatistics, Mayo Clinic, Rochester, MN 55905, United States; Division of Hematology/Oncology, Mayo Clinic Cancer Center, Mayo Clinic, Phoenix, AZ, United States; Division of Hematology/Oncology, Mayo Clinic Cancer Center, Mayo Clinic, Phoenix, AZ, United States

**Keywords:** gastrointestinal cancer, FDA approvals, surrogate endpoints, accelerated approvals, ESMO-MCBS

## Abstract

**Background:**

The increasing reliance on accelerated approvals and surrogate endpoints for Food and Drug Administration (FDA) approvals of gastrointestinal (GI) cancer therapies raises concerns about their clinical benefit and long-term patient outcomes. The shift toward single-arm trials in regulatory decisions further complicates treatment evaluation.

**Material and Methods:**

This retrospective observational study evaluated all FDA approvals for GI cancer therapies from January 2006 through January 2025. Data were extracted from FDA archives, ClinicalTrials.gov, and PubMed. Approvals were categorized by regulatory pathway (accelerated vs regular), trial design (single-arm vs randomized), and primary endpoint (surrogate vs overall survival [OS]). Clinical benefit was assessed based on OS improvement and the European Society for Medical Oncology Magnitude of Clinical Benefit Scale (ESMO-MCBS). The primary outcome was the proportion of approvals based on surrogate versus OS endpoints. Secondary outcomes included use of single-arm designs, frequency of accelerated approvals, OS gain, and the proportion meeting ESMO-MCBS substantial benefit (score ≥ 4).

**Results:**

The FDA granted 60 GI cancer drug approvals from 67 trials. Approvals rose from 15 (25%) in 2006-2014 to 45 (75%) in 2015-2025. Single-arm trials increased to 24%, and surrogate endpoints were used in 41.8% (ORR 20.9%, PFS 19.4%). Median OS improvement was 2.1 months (IQR: 1.6-2.65). Only 24.3% of trials met ESMO-MCBS substantial benefit. Of 15 accelerated approvals, 66.7% remained pending, 13.3% received full approval, and 20% were withdrawn.

**Conclusion:**

Expedited approvals have improved drug access in GI oncology, but modest benefits highlight the need to balance speed with outcomes that truly matter to patients.

Implications for practice Many recent FDA approvals for GI cancer drugs have used accelerated pathways, relying on early results like response rate or single-arm trials instead of stronger evidence from randomized studies. While this can get treatments to patients sooner, the actual benefit in survival is often limited. This study helps oncologists understand the trade-off between faster access and proven outcomes, encouraging more thoughtful discussions with patients. It also points to the need for a better balance in the approval process, one that supports timely access but ensures the treatments truly make a meaningful difference.

## Introduction

There has been an increase in the number of anticancer drugs that have been approved by the Food and Drug Administration (FDA).^1^ The primary aim of these treatments is to extend life expectancy and improve quality of life (QoL), typically assessed by overall survival (OS) or QoL metrics.^2^ In addition to approvals based on OS, the FDA grants approvals based on surrogate endpoints such as progression-free survival (PFS) and objective response rate (ORR).^3^ While these surrogates expedite drug availability, their correlation with OS is often weak, raising concerns about their clinical relevance.^3^^,^^4^

The use of surrogate endpoints in this pathway has facilitated earlier market availability of drugs.^5^^,^^6^ However, the terms “unmet needs” and “outstanding treatment” lack a standardized definition, which can make their interpretation challenging. In addition, when a trial is approved based on the accelerated pathway, the FDA mandates postmarketing trials proving the efficacy of the treatment, with the possibility of withdrawal of the treatment if the subsequent trial was never done or showed a higher risk to benefit ratio.^7^ Unfortunately, significant delays in postmarketing studies are common.^4^

In addition to the challenges associated with approvals based on surrogate endpoints, the clinical benefit of approved drugs varies across different cancers. A study by Jiang et al. found that, between 2006 and 2017, only 18% of FDA approved gastrointestinal (GI) cancer treatment had substantial clinical benefit. Since then, nearly 44 more approvals were granted by the FDA for the GI oncology field.

In addition, despite their clinical benefit, these treatments—particularly targeted therapy and immunotherapy—incur substantial costs.^8^ Therefore, an updated assessment of the studies leading to FDA approvals, along with their clinical benefit, is critical for informed decision-making in both clinical practice and cancer policy. In this study, we comprehensively analyzed FDA-approved GI cancer treatments over the past two decades, focusing on approval pathways, the use of surrogate endpoints, and their clinical benefit.

## Methods

In this study, we sought to evaluate the trend of FDA approvals in GI cancers over the past two decades, and the studies that led to these approvals. This study utilized publicly accessible data sources and did not involve human subjects; thus, no institutional review board approval was needed.

### Data identification

The FDA website was searched for anticancer drugs approved in GI malignancies from January 2006 through January 2025.^1^ January 2006 was chosen as it was the earliest date for which approval data were available on the FDA website. The search was conducted on January 31, 2025. For each approval, the following information was extracted: date, pathway (accelerated vs regular), indication, associated labels, and whether an approval was based on primary or secondary endpoint. For trials that initially received accelerated approval and were later converted to full approval, we classified them according to their status at the time of the initial FDA decision. The class of therapy for each approved drug was sourced from the National Cancer Institute (NCI) Drug Dictionary (Cancer.gov).^9^

Studies cited in each drug label as the basis of the FDA approval were identified and searched on ClinicalTrials.gov and PubMed. Trials’ articles were identified through PubMed, and protocols were reviewed if available in the Supplementary Materials or from ClinicalTrials.gov. For each trial, data pertaining to the primary and secondary endpoint(s), experimental and control arm, and patient demographics were extracted. When available, updated data on efficacy and QoL were also included.

### Evaluating clinical benefit

The clinical benefit of each trial was quantified using the European Society for Medical Oncology Magnitude of Clinical Benefit Scale (ESMO-MCBS) version1.1 2017.^7^ The tool assigns trials in the curative setting a grade of A, B, or C, with A as the highest possible grade. For trials with non-curative indications, the tool assigns a score ranging from 1 to 5, with higher scores indicating greater clinical benefit. ESMO-MCBS assesses clinical benefit based on the lower limit of the 95% confidence interval (CI) for the hazard ratio (HR) and the absolute difference in outcomes, while using toxicity and QoL as modifying factors. Two independent authors (F.J. and O.S.) scored the trials using the ESMO-MCBS evaluations forms, with a third author (MBS) resolving any disagreement. Substantial clinical benefit was defined as an ESMO-MCBS score of 4 or 5 for trials following the non-curative form and A or B for those following the curative form.

### Data analysis

To evaluate the changes of FDA approvals over time, if any, we split the study period into two equal periods: January 2006-December 2014, and January 2015-January 2025. The analysis was descriptive. Proportions, means, medians, and ranges were reported where appropriate. The graphics were generated by Microsoft Excel functions and PowerPoint.

## Results

Between January 2006 and January 2025, the FDA granted 60 approvals in GI anticancer drugs based on 67 trials. In the period of January 2006-December 2014, the FDA approved 15 drugs (25%) based on 17 clinical trials (25.4%), while the later period of January 2015-January 2025 accounted for 45 drug approvals (75%) from 50 clinical trials (74.6%). Targeted therapy studies were the most common (29/67; 43.3%), followed by immunotherapy (19/67; 28.4%) and cytotoxic chemotherapy (5/67; 7.5%). Additionally, there were 13 trials for combination therapy (19.4%), and one trial involving a radiopharmaceutical therapy (177Lu-DOTATATE in the NETTER-1 trial) classified as ‘other’ (1.5%; Table 1 and Table S1).

**Table 1.** Characteristics of trials that led to GI anticancer drugs approval between 1/2006 and 2/2025.

	Earlier period (01/2006-12/2014)	Later period (01/2015-01/2025)	Overall
Trial characteristics	*n* (%)	*n* (%)	*n* (%)
** Number of trials**	17 (25.4)	50 (74.6) 67	
**Cancer location**			
** CRC**	9 (52.9)	12 (24)	21 (31.3)
** Gastroesophageal**	3 (17.6)	15 (30)	18 (26.9)
** Pancreatic**	1 (5.9)	3 (6)	4 (6)
** Biliary**	0	7 (14)	7 (10.4)
** HCC**	1 (5.9)	11 (22)	12 (17.9)
** NEN**	3 (17.6)	2 (4)	5 (7.5)
**Classes of therapies**			
** Targeted Therapy**	10 (58.8)	19 (38)	29 (43.3)
** Immunotherapy**	0	19 (38)	19 (28.4)
** Cytotoxic chemotherapy**	1 (5.9)	4 (8)	5 (7.5)
** Combined**	6 (35.3)	7 (14)	13 (19.4)
** Other**	0 (0)	1 (2)	1 (1.5)
**Type of trial**			
** Phase II single arm**	0	12 (24)	12 (17.9)
** Phase II RCT**	0	1 (2)	1 (1.5)
** Phase III RCT**	17 (100)	37 (74)	54 (80.6)
**Primary endpoint**			
** OS**	11 (64.7)	19 (38)	30 (44.8)
** PFS**	6 (35.3)	7 (14)	13 (19.4)
** ORR**	0	14 (28)	14 (20.9)
** Multiple Primary Endpoints (OS and PFS)**	0	9 (18)	9 (13.4)
** DFS**	0	1 (2)	1 (1.5)
**Reported significant benefit in primary/multiple primary endpoints (OS and PFS)^a^**	10 (90.9)	24 (85.7)	34 (87.2)
**Median of median OS increase in months (range)**			
** CRC**	1.5 (0.3-2.1)	3 (1.6-40.8)	1.9 (0.3-40.8)
** Gastroesophageal**	2.2 (1.4-2.7)	2.3 (0.8-3.6)	2.2 (0.8-3.6)
** Pancreatic**	2.1 (single trial)	2 (1.9-2)	2 (1.9-2.1)
** Biliary**	N/A	1.7 (1.6-1.8)	1.7 (1.6-1.8)
** HCC**	2.8 (single trial)	2 (1.2-5.8)	2.2 (1.2-5.8)
** NEN**	N/A	N/A	N/A
**ESMO-MCBS score^b^**			
** 1**	5 (29.4)	8 (16.3)	13 (19.7)
** 2**	4 (23.5)	11 (22.4)	15 (22.7)
** 3**	5 (29.4)	17 (34.7)	22 (33.3)
** 4**	2 (11.8)	8 (16.3)	10 (15.2)
** 5**	1 (5.9)	5 (10.2)	6 (9.1)

a The denominator in the “Reported significant benefit in primary/multiple primary endpoints (OS and PFS)” row represents the total number of trials that included OS as a primary endpoint or as one of multiple primary endpoints.

b The total ESMO-MCBS scores for the trials summed to 66 because the two trials following the curative approach were excluded from the calculation, and KEYNOTE-811 was assigned two separate ESMO-MCBS scores.

Abbreviations: CRC: colorectal cancer, DFS: disease-free survival, ESMO-MCBS: European Society for Medical Oncology—Magnitude of Clinical Benefit Scale, HCC: hepatocellular carcinoma, NEN: neuroendocrine neoplasm, ORR: objective response rate, OS: overall survival, PFS: progression-free survival, RCT: randomized clinical trial.

### Phase of trial

Overall, most approvals were based on phase III randomized controlled trials (RCTs) (54/67; 80.6%). This was consistent over time with phase III accounting for all approvals in the earlier period (17/17; 100%) and the majority of approvals in the later period (37/50; 74%). In contrast, all the phase II single-arm studies which resulted in approvals were in the later period, accounting for 12 of 50 trials (24%; Table 1).

### Pathway of approvals

**Figure 1.**
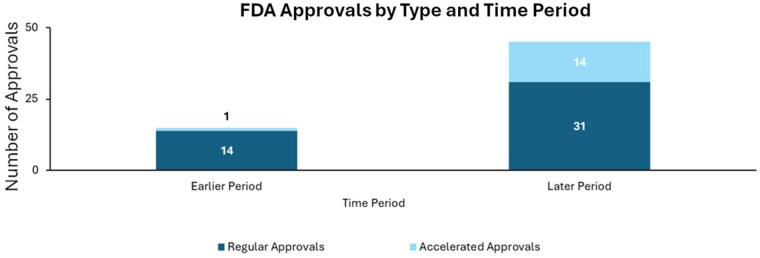
Pathway of approval across both periods. Earlier period: 01/2006-12/2014, Later period: 01/2015-01/2025.

**Figure 2.**
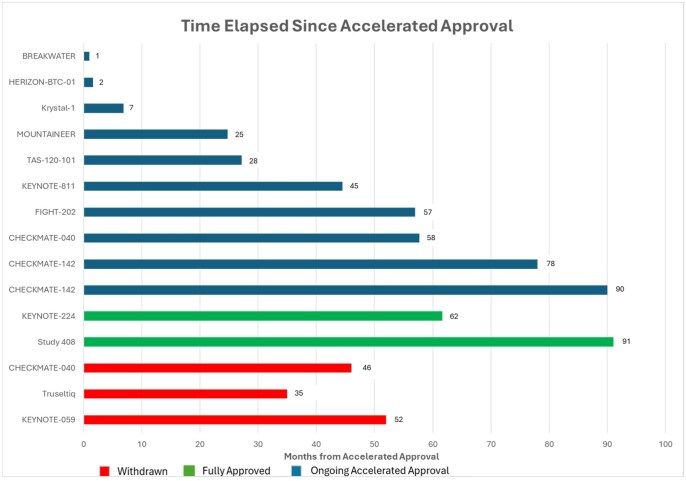
Time elapsed since accelerated approval.

Of the 60 FDA approvals granted during the study period, 45 (75%) were full approvals, while 15 (25%) were accelerated approvals. Accelerated approvals were predominantly seen in the later period, accounting for 14 of the 15 accelerated approvals (93.3%; Figure 1). In contrast, only one accelerated approval (6.7%) was granted in the earlier period, which eventually received full approval.^10^ Of the 14 accelerated approvals in the later period, 10 (71.4%) were still pending full approval at the time of analysis, one (7.1%) had already received full approval, and three (21.4%) had been withdrawn. The reasons for withdrawal varied. For example, infigratinib was granted an accelerated approval in May 2021 for advanced cholangiocarcinoma with fibroblast growth factor receptor 2 (FGFR2) alterations based on a single-arm study showing ORR of 23%.^11^^,^^12^ The approval required the sponsor to conduct postmarketing trials to verify the drug’s clinical benefit. However, the sponsor voluntarily requested withdrawal of infigratinib given difficulties enrolling patients in confirmatory clinical trials in the first line setting.^12^^,^^13^ Similarly, nivolumab received accelerated approval for patients with hepatocellular carcinoma (HCC) who have been previously treated with sorafenib based on CHECKMATE-040 study showing an ORR of 14.3%.^14^^,^^15^ However, the subsequent negative study (CHECKMATE-459) of nivolumab vs sorafenib led the sponsor to withdraw the indication of nivolumab.^13^^,^^16^ Additionally, pembrolizumab was granted accelerated approval for recurrent locally advanced or metastatic gastric and gastroesophageal junction (GEJ) adenocarcinoma whose tumors express programmed death ligand-1 (PD-L1) following the KEYNOTE-059 trial.^17^^,^^18^ However, given the negative subsequent studies of KEYNOTE-061 (second-line) and KEYNOTE-062 (first-line), the sponsor withdrew the approval (Figure 2).^13^^,^^19^^,^^20^

### Endpoints of trial

Overall, the most common primary endpoint across the 67 trials was OS (30/67; 44.8%). Dual primary endpoints of OS and PFS were employed in 9 trials (13.4%). Surrogate endpoints were used in 28 trials (41.8%), of which PFS was used in 13 trials (19.4%), ORR was used in 14 trials (20.9%), and disease-free survival (DFS) was used in one trial (1.5%). In the earlier period, OS was the most common primary endpoint with 11 trials (64.7%), followed by PFS (*n* = 6, 35.3%). In the later period, ORR became prominent, serving as the primary endpoint in 14 trials (28%), while OS remained the most common (*n* = 19 trials; 38%), and the use of PFS as a primary endpoint decreased to less than 15% (7/50). Of the 60 approvals, the majority were granted based on primary endpoints, with the exception of five approvals in the later period (Checkmate 648 treatment group 1, Checkmate 648 treatment group 2, Checkmate 649, BEACON, and Keynote 811) that were granted based on secondary endpoints.^21–28^ These approvals were considered secondary endpoint-based because they were granted based on an endpoint not defined as primary or extrapolated to a population not included in the primary endpoint (Table 2).

**Table 2.** Overview of FDA approvals for oncology drugs based on secondary endpoints.

Study name	Approved drug	Indication	Primary endpoint(s)	Endpoint used for FDA approval
**Checkmate-648 (cohort 2)**	Nivolumab with Fluoropyrimidine and platinum-based chemotherapy.	Advanced or metastatic ESCC.	OS in participants with tumor cell PD-L1 of 1% or greater PFS as assessed by BICR in participants with tumor cell PD-L1 of 1% or greater.	Full approval: OS in all randomized participants.
**Checkmate-648 (cohort 1)**	Nivolumab with Ipilimumab.	Advanced or metastatic ESCC.	OS in participants with tumor cell PD-L1 of 1% or greater PFS as assessed by BICR in participants with tumor cell PD-L1 of 1% or greater.	Full approval: OS in all randomized participants.
**Keynote-811**	Pembrolizumab with Trastuzumab, Fluoropyrimidine, and platinum containing chemotherapy.	Locally advanced unresectable or metastatic HER2 positive gastric or GEJ adenocarcinoma.	OS. PFS per RECIST 1.1 assessed by BICR.	Accelerated approval on 05/05/2021: ORR per RECIST 1.1 assessed by BICR based on interim analysis regardless of the PDL1. On Nov 7, 2023: amendment of the accelerated approval to be only in PDL1 CPS of 1 or above
**Checkmate-649**	Nivolumab with Fluoropyrimidine and platinum containing chemotherapy.	Advanced or metastatic gastric cancer, GEJ cancer, and esophageal adenocarcinoma.	OS in participants treated with Nivolumab Plus Chemotherapy vs Chemotherapy with PD-L1 CPS ≥ 5. PFS in participants treated with Nivolumab Plus Chemotherapy vs Chemotherapy With PD-L1 CPS ≥ 5.	OS in all participants treated with Nivolumab plus Chemotherapy vs Chemotherapy.
**BEACON**	Encorafenib with Cetuximab.	Metastatic CRC with a BRAF V600E mutation.	OS of triplet arm vs control arm—interim analysis (Phase 3). ORR by BICR Per RECIST, v1.1 of triplet Arm vs control Arm.	OS in doublet arm vs control arm.

Abbreviations: BICR: blinded independent central review, CPS: combined positive score, CRC: colorectal carcinoma, ESCC: esophageal squamous cell carcinoma, GEJ: gastroesophageal junction, ORR: objective response rate, OS: overall survival, PD-L1: programmed death ligand 1, PFS: progression free survival, RECIST: response evaluation criteria in solid tumors.

### Survival outcomes and QoL

Among the 39/67 trials (58.2%) that reported OS as a primary endpoint, either exclusively or as multiple primary endpoints, the median of the median OS increase was 2.1 months (interquartile range: 1.6-2.65; range: 0.3-40.8; Table S1). When stratified by cancer type, the median of the median OS increase did not exceed 2.2 months in any subgroup (Table 1). In terms of QoL outcomes in these trials with OS as primary endpoint, only 12 out of the 39 trials (30.8%) reported an improvement. Most trials (23/39, 59%) showed no improvement in QoL, while the remaining 4 trials (10.3%) did not publish any QoL data.

Of the trials that utilized surrogate endpoints as their primary outcome (*n* = 28), 11 trials (11/28; 39.3%) demonstrated an improvement in OS with seven (25%) of them reaching statistical significance. QoL was assessed in 75% (21/28) of studies utilizing surrogate endpoints, with improvement observed only in five (17.9%) trials.

### ESMO magnitude of clinical benefit scale

Among the 67 trials that led to approvals, two trials were graded based on the curative ESMO-MCBS forms (CHECKMATE-577 and KEYNOTE-590), while the other 65 trials were graded based on non-curative ESMO-MCBS forms. CHECKMATE-577 followed the curative form because the study was done in a curative setting.^29^ In contrast, KEYNOTE-590 followed a similar form per ESMO-MCBS recommendation because the 5-year data continued to demonstrate OS survival benefit.^30^ Among the trials that followed the noncurative ESMO-MCBS forms, 16 trials (24.3%) met the ESMO-MCBS criteria for substantial clinical benefit (grades 4-5). In the earlier period, 17.7% (3/17) of trials met the ESMO-MCBS criteria for substantial clinical benefit compared to 26.5% (13/49) in the later period. Keynote-811 received two ESMO-MCBS because the original and the updated approvals were based on different endpoints (Table S1).

### Demographics

Across all trials, the elderly was consistently defined as individuals aged 65 years or older. Of the 67 trials, 37 (55.2%) reported the proportion of elderly participants, with an average of approximately 39.9%, ranging from 14% to 58%. Of the 67 trials, 36 (53.7%) provided the exact percentage of participants from the United States or North America. Among these, 29 trials (80.6%) indicated that less than 50% of their participants were from the United States or North America, while 15 trials (41.7%) had less than 15%, and 11 trials (30.6%) had less than 10%. With respect to ethnicity, 49 trials reported the exact proportion of White participants, with a median of 61% (range 4-98.5), while 45 trials reported the proportion of African American participants, with a median of 2% (range 0-13.8).

## Discussion

In this study, we analyzed FDA-approved GI cancer treatments from January 2006 through January 2025 looking at approval pathways, trial designs, and clinical benefit. We observed an increase in accelerated approvals after the year 2015, with a rise in approvals based on single-arm phase II trials. OS remained the most used endpoint, with the median of the median OS increase of 2.1 months, among trials that used it as a primary or multiple primary endpoints. Furthermore, 24.3% of the trials demonstrated substantial clinical benefit based on ESMO-MCBS.

Selecting an appropriate primary endpoint is critical in clinical trial design, as it determines the clinical relevance of a drug’s benefit.^31^ Patients seek anticancer treatment that helps increase their OS and improve their QoL.^32^ We found that nearly half of GI cancer trials from January 2006 to January 2025 relied on surrogate endpoints rather than OS, mirroring trends seen across various cancer types.^33^ Furthermore, OS benefit was limited to ≤6 months in all trials except KEYNOTE-177. This pattern is not unique to GI cancers; Iskander et al reported a median OS gain of only 1.18 months across various cancer types between 2017 and 2021, reinforcing concerns about the modest survival benefits associated with many newly approved therapies.^34^ According to the American Society of Clinical Oncology (ASCO), an OS improvement of at least 2.5-6 months (depending on baseline prognosis) is considered clinically meaningful, which underscores that the median OS gain we observed (2.1 months) falls below this threshold.^35^

The increasing reliance on surrogate endpoints in GI cancer trials reflects a broader trend across oncology.^33^ Between January 2006 and January 2025, surrogate endpoints were used in 41.8% of GI cancer trials, with a consistent pattern observed in both periods. While the use of surrogate endpoints is considered appropriate in certain cases, such as PFS in neuroendocrine tumors and 3-year DFS in the adjuvant treatment of colon cancer,^36^^,^^37^ their widespread use raises concerns about their reliability in predicting meaningful clinical benefit. Additionally, surrogate endpoints such as PFS and ORR rely on radiological measurements, and frequent imaging may lead to loss to follow-up, resulting in censored data.^38^ For instance, in three major clinical trials- RADIANT-2, RADIANT-3, and RADIANT-4- everolimus demonstrated PFS benefit but failed to show OS benefit.^39^ This discrepancy could be attributed to informative censoring, as 35% of the patients who received everolimus were censored compared to 20% in the control arm.^39^ This informative censoring could be secondary to more adverse events in the everolimus arms leading to some patients being lost to follow up, or related to discrepancy in calling progression between investigator-assessed and centrally blinded assessment.^40^^,^^41^ Additionally, surrogate endpoints do not always correlate with the outcomes that matter to patients.^42^^,^^43^ Among the 28 GI cancer trials using surrogate endpoints in our study, 60.7% (*n* = 17) showed no significant OS or QoL benefit. Similarly, Paratore et al. found that when improvements in PFS as the primary endpoint without significant improvement in OS, only 20.5% of the trials show improvement in global QoL in the treatment arm.^31^ These findings highlight the need for either significant QoL and/or OS when approving medications based on surrogate endpoints.

The accelerated approval pathway was established to expedite the availability of new treatments for life-threatening conditions with unmet needs.^6^ However, the lack of formal definition of “unmet needs” creates ambiguity in approval decisions. We found an increasing trend in accelerated GI cancer treatment approvals, with 31.1% occurring after 2015 compared to only 6.7% before 2015.^1^ Unlike regular approvals, accelerated approvals require postmarketing surveillance,^44^ and the FDA has the authority to withdraw a drug if confirmatory trials are not conducted or fail to demonstrate benefits.^6^ While awaiting the confirmatory trials, patients may be subjected to unnecessary toxicities from cancer treatments that are approved under the accelerated pathway. We found that there was a period of 35-52 months to confirm the need to withdraw three of the already approved GI cancer medications.^13^ In addition, there is often a significant delay between the FDA’s withdrawal of a drug and the update of the National Comprehensive Cancer Network (NCCN) guidelines.^45–47^ For instance, nivolumab was withdrawn for HCC on July 23, 2021, but remained listed as a category 2A recommendation in NCCN guidelines until October 14, 2022.^45^ This delay is important because keeping withdrawn drugs in clinical guidelines can influence prescribing decisions and payer coverage, underscoring the need for timely updates. Furthermore, accelerated approvals and continued NCCN listing of unconfirmed treatments may also hinder enrollment in ongoing clinical trials investigating more effective therapies.

It is important to recognize the increasing trend in GI cancer studies where results are being reported focusing only on the nested subgroups (PD-L1 ≥ 5), without reporting outcomes for adjacent subgroups (outcomes for PD-L1 negative vs 1-5 vs ≥5). For instance, CHECKMATE-649 demonstrated a significant OS benefit of adding nivolumab to chemotherapy in patients with unresectable GEJ and gastric cancer in PD-L1 combined positive score (CPS) ≥ 1 nested subgroup, the PD-L1 CPS ≥ 5 nested subgroup, and all comers, leading to the approval of the regimen for all patients regardless of the PD-L1 level.^26^^,^^48^ However, no OS benefit was observed in patients with PD-L1 CPS < 5 (OS: 12.4 vs 12.3 months, HR: 0.95 [0.8-1.12]).^49^ A similar pattern was observed in two other trials, KEYNOTE-859 and RATIONAL 306.^50^^,^^51^ In September 2024, the Oncologic Drugs Advisory Committee voted against the use of checkpoint inhibitors in HER2-negative gastric/GEJ adenocarcinoma with PD-L1 expression less than 1 based on pooled analysis done by the FDA showing lack of benefit in this patient population.^52^

A previous study (2006-2017) reported that only 18% of FDA-approved GI cancer treatments met the ESMO-MCBS substantial clinical benefit threshold (score 4-5).^8^ We found a similar trend, with 24.3% (*n* = 16) of GI cancer trials demonstrating substantial clinical benefit. However, in the United States, FDA approval is not dependent on meeting these value thresholds, and reimbursement or guideline adoption decisions are often not aligned with ESMO-MCBS scores. This disconnect highlights the potential for widespread clinical adoption of therapies with only modest benefit, raising questions about cost-effectiveness and patient access to truly impactful treatments. At the same time, ESMO-MCBS has limitations, including lack of trial sample size consideration, incomplete toxicity adjustment, and the exclusion of cost analysis.^8^ It is also important to note that different value frameworks may lead to different assessments of the same therapy. For instance, the ASCO Value Framework formally integrates clinical benefit, cost and toxicity, and includes additional consideration for durable survival or symptom relief- factors that are not fully captured by ESMO-MCBS. Moreover, while ESMO-MCBS is geared toward health policy and reimbursement discussions, the ASCO framework is intended for individualized clinical decision-making, which may lead to divergent evaluations of the same drug.^53^

We also assessed the representation of North American populations in GI cancer trials. The FDA does not mandate a specific cutoff for the inclusion of North American or U.S. populations, race, or ethnicity in clinical trials for drug approval. However, it encourages diversity in clinical trial populations, including geographic representation.^54^ Among the trials analyzed, 53.7% (*n* = 36) reported the proportion of North American or U.S. participants. Of these, 30.6% (11/36) had fewer than 10% of patients from these regions, raising concerns about the generalizability of trial findings to North American populations.

This study offers a comprehensive evaluation of FDA approval trends in GI oncology over the past two decades, offering valuable insights for future regulatory and clinical decision-making. The use of ESMO-MCBS, a reproducible and systematic tool, adds rigors to the evaluation of clinical benefit. However, a few limitations should be acknowledged. The study relied on publicly available data, which might result in incomplete information. Our focus on FDA approvals, may not fully capture the benefits of GI cancer treatments globally. While cost analysis was not included in this study, prior studies had looked into this with median total drug price of $62 415 and median monthly average wholesale price of $14 769 per patients, with a range from $6531 to $20 764.^8^

## Conclusion

This study underscores a shift in FDA approvals for GI cancer treatments over the past two decades, with a growing reliance on accelerated approvals and single-arm studies. While these trends have expedited drug availability, they have often resulted in treatments with modest clinical benefit and delayed the confirmatory trial conduct. Moving forward, regulatory agencies should balance overall-survival, quality of life, cost, and the need for expedited drug access to ensure that newly approved therapies align with meaningful patient outcomes. Strengthening postmarketing requirements and ensuring timely confirmatory trials will be essential to uphold the integrity of the approval process and patient-centered cancer care.

## Supplementary Material

oyaf381_Supplementary_Data

## Data Availability

The data underlying this article are available in the article and in its online supplementary material.
