# Supplementary material for: Evaluating Food and Drug Administration approved gastrointestinal cancer drugs: clinical benefit and trial endpoints over the past two decades
Source: Oncologist. 2025 Dec 8;30(12):oyaf381. doi: 10.1093/oncolo/oyaf381 (PMC12693570; doi:10.1093/oncolo/oyaf381)
Supplement: oyaf381_Supplementary_Data [file oyaf381_supplementary_data.zip › Supplement (1).docx]

| Cancer Location | Clinical Trial Number | Trial Name | Phase upon Approval | Treatment | FDA Approval Year | Reason for FDA Approval | Primary Endpoint | OS and PFS benefit-experimental arm(mo)  (If reported) | Absolute gain in OS and PFS (if RCT) (mo) | QoL Benefit | ESMO-MCBS |
| --- | --- | --- | --- | --- | --- | --- | --- | --- | --- | --- | --- |
| Colorectal | NCT06252649 | CodeBreaK 301 | 3 | sotorasib with panitumumab | 1/16/2025 | ↑ in PFS | PFS | OS: NE  PFS: 5.6 | OS: N/A  PFS: 3.6 | Yes | 4 |
| Colorectal | NCT04607421 | BREAKWATER | 3 | encorafenib with cetuximab and mFOLFOX6 | 12/20/2024 | ↑ in ORR | ORR | OS: NE  PFS: N/A | OS: N/A  PFS: N/A | N/A | 2 |
| Biliary tree | NCT04466891 | HERIZON-BTC-01 | 2 | zanidatamab-hrii | 11/20/2024 | ↑ in ORR | ORR | OS: 18.1  PFS: 5.5 | N/A  Yes | Yes | 4 |
| Gastric and GEJ | NCT03653507 | GLOW | 3 | zolbetuximab-clzb with CAPOX chemotherapy | 10/18/2024 | ↑ in PFS | PFS | OS: 14.4  PFS: 8.2 | OS: 2.2  PFS: 1.4  No | No | 3 |
| Gastric and GEJ | NCT03504397 | SPOTLIGHT | 3 | zolbetuximab-clzb with fluoropyrimidine- and platinum-containing chemotherapy | 10/18/2024 | ↑ in PFS | PFS | OS: 18.2  PFS: 10.6 | OS: 2.7  PFS: 1.9  No | No | 3 |
| Colorectal | NCT03785249 | Krystal-1 | 2 | adagrasib with cetuximab | 6/21/2024 | ↑ in ORR | ORR | OS: 15.9  PFS: 6.9 | N/A | N/A | 3 |
| Pancreatic | NCT04083235 | NAPOLI 3 | 3 | NALIRIFOX | 2/13/2024 | ↑ in OS | OS | OS: 11.1  PFS: 7.4 | OS: 1.9  PFS: 1.8 | N/A | 2 |
| Gastric and GEJ | NCT03675737 | KEYNOTE-859 | 3 | pembrolizumab plus chemotherapy | 11/16/2023 | ↑ in OS | OS | OS: 12.9  PFS: 6.9 | OS: 1.4  PFS: 1.3 | No | 1 |
| Colorectal | NCT04322539 | FRESCO-2 | 3 | BSC plus fruquintiib | 11/8/2023 | ↑ in OS | OS | OS: 7.4  PFS: 3.7 | OS: 2.6  PFS: 1.9 | Yes | 4 |
| Biliary tree | NCT04003636 | KEYNOTE-966 | 3 | pembrolizumab plus gemcetabine with cisplatin | 10/31/2023 | ↑ in OS | OS | OS: 12.7  PFS: 6.5 | OS: 1.8  PFS: 0.9 | No | 1 |
| Colorectal | NCT04737187 | SUNLIGHT | 3 | bevacizumab plus FTD-TPI | 8/2/2023 | ↑ in OS and PFS | OS | OS: 10.8  PFS: 5.6 | OS: 3.3  PFS: 3.2 | No | 4 |
| Gastric and GEJ | NCT03615326 | KEYNOTE-811 (this is not an approval but a continuation of the trial) | 3 | pembrolizumab plus trastuzumab and FU and platinium based | 3/1/2023 | N/A | OS and PFS | OS: 20  PFS: 10.9 | OS: 3.2  PFS: 3.6 | No | 1 |
| Colorectal | NCT03043313 | MOUNTAINEER | 2 | tucatinib with trastuzumab | 1/19/2023 | ↑ in ORR | ORR | OS: 24.1  PFS: 8.2 | N/A | No | 3 |
| Hepatocellular | NCT03298451 | HIMALAYA | 3 | tremelimumab with durvalumab | 10/21/2022 | ↑ in OS | OS | OS: 16.4  PFS: 3.8 | OS: 2.6  PFS: -0.3 | Yes | 5 |
| Hepatocellular | NCT03062358 | KEYNOTE-394 (this is not an approval, but a subsequent trial) | 3 | pembrolizumab | 10/10/2022 | N/A | OS | OS: 14.6  PFS: 2.6 | OS: 1.3  PFS: 0.3 | Yes | 3 |
| Biliary tree | NCT02052778 | TAS-120-101 | 2 | futibatinib | 9/30/2022 | ↑ in ORR | ORR | OS: 21.7  PFS: 9 | N/A | No | 3 |
| Biliary tree | NCT03875235 | TOPAZ-1 | 3 | durvalumab with gemcitabine and cisplatin | 9/2/2022 | ↑ in OS | OS | OS: 12.9  PFS: 7.2 | OS: 1.6  PFS: 1.5 | No | 2 |
| Esophageal | NCT03143153 | CHECKMATE-648 | 3 | nivolumab with 5FU and cisplatin | 5/27/2022 | ↑ in OS and PFS | OS and PFS | OS: 13.2  PFS: 5.8 | OS: 2.5  PFS: 0.2 | Yes | 4 |
| Esophageal | NCT03143153 | CHECKMATE-648 | 3 | nivolumab with ipilimumab | 5/27/2022 | ↑ in OS | OS and PFS | OS: 12.7  PFS: 2.9 | OS: 2  PFS: -2.7 | Yes | 4 |
| Biliary tree | NCT02989857 | Study AG120-C-005 (ClarIDHy) | 3 | ivosidenib | 8/25/2021 | ↑ in PFS | PFS | OS: 10.3  PFS: 2.7 | OS: 2.8  PFS: 1.3 | No | 2 |
| Biliary tree | NCT02150967 | TRUSELTIQ | 2 | infigratinib | 5/28/2021 | ↑ in ORR | ORR | OS: 12.2  PFS: 7.3 | N/A | N/A | 3 |
| Esophageal and GEJ | NCT02743494 | CHECKMATE-577 | 3 | nivolumab | 5/20/2021 | ↑ in DFS | DFS | OS: NR  DFS:22.4 | OS: NR  DFS:11.4 | No | B |
| Gastric and GEJ | NCT03615326 | KEYNOTE-811 | 3 | pembrolizumab plus trastuzumab and FU and platinium based | 5/5/2021 | ↑ in ORR | OS and PFS | OS: 20  PFS: 10.9 | OS: 3.2  PFS: 3.6 | No | 2 |
| Gastric and esophageal | NCT02872116 | CHECKMATE-649 | 3 | nivolumab with FOLFOX or XELOX | 4/16/2021 | ↑ in OS and PFS | OS and PFS | OS: 13.8  PFS: 7.7 | OS: 2.2  PFS: 0.8 | Yes | 3 |
| Esophageal and GEJ | NCT03189719 | KEYNOTE-590 | 3 | pembrolizumab with cisplatin and FU | 3/22/2021 | ↑ in OS and PFS | OS and PFS | OS: 12.4  PFS: 6.3 | OS: 2.6  PFS: 0.5 | No | A/3 |
| Gastric and GEJ | NCT03329690 | DESTINY-Gastric01 | 2 | fam-trastuzumab deruxtecan-nxki | 1/15/2021 | ↑ in OS and ORR | ORR | OS: 12.5  PFS: 5.6 | OS: 4.1  PFS: 2.1 | Yes | 2 |
| Colorectal | NCT02563002 | KEYNOTE-177 | 3 | pembrolizumab | 6/29/2020 | ↑ in PFS | OS and PFS | OS: 77.5  PFS: 36.7 | OS:40.8  PFS: 8.3 | Yes | 5 |
| Esophageal | NCT02569242 | ATTRACTION-3 | 3 | nivolumab | 6/10/2020 | ↑ in OS | OS | OS: 10.9  PFS: 1.7 | OS: 2.4  PFS: -1.7 | Yes | 4 |
| Hepatocellular | NCT03434379 | IMbrave150 | 3 | atezolizumab followed by bevacizumab | 5/29/2020 | ↑ in OS and PFS | OS and PFS | OS: 19.2  PFS: 6.9 | OS: 5.8  PFS: 2.6 | Yes | 5 |
| Biliary tree | NCT02924376 | FIGHT-202 | 2 | pemigatinib | 4/17/2020 | ↑ in ORR | ORR | For FGFR fusions or rearrangement subgroup:  OS: 21.1  PFS: 6.9  For other FGF/FGFR alterations: OS: 6.7  PFS: 2.1 | N/A | No | 3 |
| Colorectal | NCT02928224 | BEACON | 3 | encorafenib with cetuximab | 4/8/2020 | ↑ in OS | OS | OS: 9.3  PFS: 4.3 | OS: 3.4  PFS: 2.8 | Yes | 5 |
| Hepatocellular | NCT01658878 | CHECKMATE-040 | 2 | nivolumab with ipilimumab | 3/10/2020 | ↑ in ORR | ORR | OS: 22.8  PFS: N/A | N/A | N/A | 3 |
| Pancreatic | NCT02184195 | POLO | 3 | olaparib | 12/27/2019 | ↑ in PFS | PFS | OS: 19  PFS: 7.4 | OS: -0.2  PFS: 3.6 | No | 2 |
| Esophageal | NCT02564263 | KEYNOTE-181 | 3 | pembrolizumab | 7/30/2019 | ↑ in OS | OS | OS: 10.3  PFS: 2.6 | OS: 3.6  PFS: -0.4 | No | 5 |
| Hepatocellular | NCT02576509 | CHECKMATE-459 (this is not an approval but a subsequent trial) | 3 | nivolumab | 6/24/2019 | N/A | OS | OS: 16.4  PFS: 3.7 | OS: 1.7  PFS: -0.1 | No | 2 |
| Hepatocellular | NCT02435433 | REACH-2 | 3 | ramucirumab with BSC | 5/10/2019 | ↑ in OS | OS | OS: 8.5  PFS: 2.8 | OS: 1.2  PFS: 1.2 | No | 1 |
| Gastric and GEJ | NCT02370498 | KEYNOTE-061 (this is not an approval but a subsequent trial) | 3 | pembrolizumab | 3/16/2019 | N/A | OS and PFS | OS: 9.1  PFS: 1.5 | OS: 0.8  PFS: -2.6 | No | 2 |
| Gastric and GEJ | NCT02500043 | TAGS | 3 | lonsurf with BSC | 2/22/2019 | ↑ in OS | OS | OS: 5.7  PFS: 2 | OS: 2.1  PFS: 0.2 | No | 3 |
| Hepatocellular | NCT01908426 | CELESTIAL | 3 | cabozantinib | 1/14/2019 | ↑ in OS | OS | OS: 10.2  PFS: 5.2 | OS: 2.2  PFS: 3.3 | No | 3 |
| Hepatocellular | NCT02702401 | KEYNOTE-240 (this is not an approval but a subsequent trial) | 3 | pembrolizumab plus BSC | 1/2/2019 | N/A | OS and PFS | OS: 13.9  PFS: 3 | OS: 3.3  PFS: 0.2 | No | 4 |
| Hepatocellular | NCT02702414 | KEYNOTE-224 | 2 | pembrolizumab | 11/9/2018 | ↑ in ORR | ORR | OS: 13.2  PFS: 4.9 | N/A | N/A | 2 |
| Hepatocellular | NCT01761266 | REFLECT | 3 | lenvatinib | 8/16/2018 | ↑ in OS | OS | OS: 13.6  PFS: 12.3 | OS: 1.3  PFS: 3.7 | No | 1 |
| Colorectal | NCT02060188 | CHECKMATE-142 | 2 | ipilimumab and nivolumab | 7/10/2018 | ↑ in ORR | ORR | OS: NE  PFS: NE | N/A | Yes | 3 |
| Gastroenteropancreatic NET | NCT01578239 | NETTER-1 | 3 | lutetium Lu 177 dotatate with long-acting octreotide | 1/26/2018 | ↑ in PFS | PFS | OS: 48  PFS: 28.4 | OS: 11.7  PFS: 20 | Yes | 3 |
| Gastric and GEJ | NCT02335411 | KEYNOTE-059 | 2 | pembrolizumab | 9/22/2017 | ↑ in ORR | ORR | OS: 8  PFS: 2 | N/A | N/A | 1 |
| Hepatocellular | NCT 01658878 | CHECKMATE-040 | 2 | nivolumab | 9/22/2017 | ↑ in ORR | ORR | OS: 15.1  PFS: NR | N/A | No | 1 |
| Colorectal | NCT 02060188 | CHECKMATE-142 | 2 | nivolumab | 8/1/2017 | ↑ in ORR | ORR | OS: Not reached  PFS: Not reached | N/A | No | 3 |
| NET of GI | NCT01578239 | RADIANT-4 | 3 | everolimus | 2/26/2016 | ↑ in PFS | PFS | OS: NE  PFS: 11 | OS: NE  PFS: 7.1 | No | 2 |
| Pancreatic | NCT01494506 | NAPOLI-1 | 3 | nanoliposomal irinotecan with FU and folinic acid | 10/22/2015 | ↑ in OS | OS | OS: 6.2  PFS: 3.1 | OS: 2  PFS: 1.6 | No | 3 |
| Colorectal | NCT01607957 | RECOURSE | 3 | trifluridine plus tipiracil with BSC | 9/22/2015 | ↑ in OS | OS | OS: 7.2  PFS: 2 | OS: 2  PFS: 0.3 | No | 3 |
| Colorectal | NCT01183780 | RAISE | 3 | FOLFIRI plus ramucirumab | 4/24/2015 | ↑ in OS | OS | OS: 13.3  PFS: 5.7 | OS: 1.6  PFS: 1.2 | No | 1 |
| Gastroenteropancreatic NET | NCT00353496 | CLARINET | 3 | lanreotide | 12/16/2014 | ↑ in PFS | PFS | OS: N/A  PFS: 38.5 | OS: N/A  PFS: 19.5 | No | 3 |
| Gastric and GEJ | NCT01170663 | RAINBOW | 3 | ramucirumab with paclitaxel | 11/5/2014 | ↑ in OS | OS | OS: 9.6  PFS: 4.4 | OS: 2.2  PFS: 1.5 | No | 2 |
| Gastric and GEJ | NCT00917384 | REGARD | 3 | ramucirumab plus BSC | 4/21/2014 | ↑ in OS | OS | OS: 5.2  PFS: 2.1 | OS: 1.4  PFS: 0.8 | No | 1 |
| Colorectal | NCT01001377 | ASPECCT (this is not an approval, but a subsequent trial) | 3 | panitumumab | 1/1/2014 | N/A | OS | OS: 10.2  PFS: 4.2 | OS: 0.3  PFS: -0.2 | N/A | 1 |
| Pancreatic | NCT02391662 | MPACT | 3 | *nab*-paclitaxel with gemcitabine | 9/6/2013 | ↑ in OS | OS | OS: 8.7  PFS: 5.5 | OS: 2.1  PFS: 1.8 | N/A | 3 |
| Colorectal | NCT00364013 | PRIME (this is not an approval, but a subsequent trial) | 3 | panitumumab plus FOLFOX4 | 3/22/2013 | N/A | PFS | OS: 23.8  PFS: 10 | OS: 4.4  PFS: 1.4 | No | 2 |
| Colorectal | NCT00700102 | ML18147 | 3 | bevacizumab plus chemotherapy | 1/23/2013 | ↑ in OS | OS | OS: 11.2  PFS: 5.7 | OS: 1.4  PFS: 1.6 | N/A | 1 |
| Colorectal | NCT01103323 | CORRECT | 3 | regorafenib | 9/27/2012 | ↑ in OS | OS | OS: 6.4  PFS: 1.9 | OS: 1.4  PFS: 0.2 | No | 1 |
| Colorectal | NCT00561470 | VELOUR | 3 | aflibercept plus FOLFIRI | 8/3/2012 | ↑ in OS | OS | OS: 13.8  PFS: 6.9 | OS: 1.9  PFS: 2 | No | 2 |
| Colorectal | NCT00154102 | CRYSTAL | 3 | cetuximab plus FOLFIRI | 7/9/2012 | ↑ in PFS | PFS | OS: 28.4  PFS: 11.4 | OS: 8.2  PFS: 3 | No | 4 |
| Pancreatic NET | NCT00428597 | SUN1111 | 3 | sunitinib | 5/20/2011 | ↑ in PFS | PFS | OS: 54.1  PFS: 12.9 | OS: N/A  PFS: 7.2 | No | 5 |
| Pancreatic NET | NCT00510068 | RADIANT-3 | 3 | everolimus | 5/5/2011 | ↑ in PFS | PFS | OS: 44  PFS: 11 | OS: 6.3  PFS: 6.4 | No | 2 |
| Gastric and GEJ | NCT01041404 | ToGA | 3 | trastuzumab plus chemotherapy | 10/20/2010 | ↑ in OS | OS | OS: 13.8  PFS: 6.7 | OS: 2.7  PFS: 1.2 | Yes | 4 |
| Hepatocellular | NCT00105443 | SHARP 2008 | 3 | sorafenib | 11/19/2007 | ↑ in OS | OS | OS: 10.7  PFS: 4.1 | OS: 2.8  PFS: -0.8 | No | 3 |
| Colorectal | NCT00079066 | CO.17 | 3 | cetuximab | 10/2/2007 | ↑ in OS | OS | OS: 6.1  PFS: 2 | OS: 1.5  PFS: 0.5 | Yes | 3 |
| Colorectal | NCT00113763 | Study 408 | 3 | panitumumab plus BSC | 9/27/2006 | ↑ in PFS | PFS | OS: 10  PFS: 2 | OS: 0.4  PFS: 0.2 | N/A | 1 |
| Colorectal | NCT00025337 | E3200 | 3 | FOLFOX4 + bevacizumab | 6/20/2006 | ↑ in OS | OS | OS: 12.9  PFS: 7.3 | OS: 2.1  PFS: 2.6 | N/A | 3 |

**Supplementary Table 1**: FDA Approvals and Clinical Outcomes of GI Cancer therapies from January 2006 till February 2025: Efficacy, QoL, and ESMO-MCBS Ratings

BSC: best standard of care, ESMO-MCBS: European Society for Medical Oncology – Magnitude of Clinical Benefit Scale, FU: fluorouracil, GEJ: gastroesophageal junction, GI: Gastrointestinal, mo: months, N/A: not applicable, NE: not evaluable, NET: neuroendocrine tumor, ORR: objective response rate, OS: overall survival, PFS: progression free survival, QoL: quality of life, RCT: randomized clinical trial, ↑: improvement.
